# Patients’ and providers’ perspectives on e-health applications designed for self-care in association with surgery – a scoping review

**DOI:** 10.1186/s12913-022-07718-8

**Published:** 2022-03-23

**Authors:** Lotta Wikström, Kristina Schildmeijer, Elisabeth Mueller Nylander, Kerstin Eriksson

**Affiliations:** 1 School of Health and Welfare, Jönköping University, Jönköping, Sweden; 2 Department of Anaesthesia and Intensive Care, Ryhov County Hospital, Jönköping, Sweden; 3 Department of Nursing Science, School of Health and Welfare, Lotta Wikström, Jönköping University, Box 1026, 551 11 Jönköping, Sweden; 4 Faculty of Health and Life Sciences, Linnaeus University, Växjö, Sweden; 5 Jönköping, Sweden

**Keywords:** e-health, Preoperative preparation, Patients, Providers, Self-care, Surgery, Postoperative recovery

## Abstract

**Background:**

Before and after major surgery, access to information in a user-friendly way is a prerequisite for patients to feel confident in taking on the responsibility for their surgical preparation and recovery. Several e-health applications have been developed to support patients perioperatively. The aim of this review was to give an overview of e-health applications designed for self-care associated with surgery by providing a scoping overview of perspectives from providers and patients.

**Methods:**

We searched the following data sources to identify peer-reviewed quantitative and qualitative studies published between 2015 and 2020: CINAHL, Google Scholar, MEDLINE, PsycInfo, Web of Science, and Scopus. After identifying 960 titles, we screened 638 abstracts, of which 72 were screened in full text. Protocol register: 10.17605/OSF.IO/R3QND.

**Results:**

We included 15 studies which met our inclusion criteria. Data from several surgical contexts revealed that the most common self-care actions in e-health applications were preoperative preparations and self-assessments of postoperative recovery. Motivational factors for self-care were information, combined with supportive reminders and messages, and chat features. Although there was great variance in research designs and technical solutions, a willingness to engage with and adhere to e-health seemed to increase patients’ self-care activities and thereby accelerate return to work and normal activities. In addition, the need for physical visits seemed to decrease. Even though age groups were not primarily studied, the included studies showed that adult patients of any age engaged in surgical self-care supported by e-health. The providers’ perspectives were not found.

**Conclusions:**

E-health applications supporting perioperative self-care indicated a positive impact on recovery. However, experiences of healthcare professionals delivering e-health associated with surgery are missing. Additionally, studies based on patients’ perspectives regarding willingness, adherence, and motivation for self-care supported by e-health are sparse. A need for studies examining the supporting role of e-health for self-care in the surgical context is therefore needed.

**Supplementary Information:**

The online version contains supplementary material available at 10.1186/s12913-022-07718-8.

## Introduction

The length of a hospital stay after major surgery has in recent years been shortened by several days [1, 2], leaving patients to take responsibility for their own recovery once discharged. User-friendly access to information is a prerequisite for patients to feel confident in taking on the responsibility for their recovery. For this reason, several e-health applications have been developed over the last two decades to support patients [3–5]. A research group mapping the concept of e-health defined it as follows:

“E-health is an emerging field of medical informatics, referring to the organization and delivery of health services and information using the internet and related technologies. In a broader sense, the term characterizes not only a technical development, but also a new way of working, an attitude, and a commitment for networked, global thinking, to improve health care locally, regionally, and worldwide by using information and communication technology” [6].

Van der Meij et al. found that the purpose of most developed e-health applications is to complement or replace standard care [7]. In addition to information, there are some applications that also give patients opportunities to report symptom experiences and vital parameters, such as pulse or blood pressure. Some e-health applications have added alerts for the purpose of making healthcare professionals aware if their patients have been severely impacted by symptom experiences [8].

The rapid development of e-health applications has led to a need for mapping different areas of use in the context of perioperative care. Six reviews were found that focused on different aspects of exchanging information between healthcare professionals and patients [7, 9–13]. Of these reviews, two have a scoping approach and four have a systematic approach. The intention of scoping reviews is to capture knowledge in novel areas according to comprehensive wide-ranging aims, while systematic reviews are to get answers to specified questions [14]. The scoping review performed by Koutras et al. explored the socioeconomic impact of e-health in perioperative care [9], while Maramba et al. examined methods to test the functionality of e-health applications [11]. Three systematic reviews examined the use of smartphone and tablet devices from an organisational perspective in the context of surgery [7, 10, 12], while one offered a patient perspective [13]. None of these reviews covered motivating and supporting effects from e-health applications in pre- and postoperative self-care.

The concept of self-care is described as taking responsibility for one’s well-being during the phase of acute illness, in contrast to the concept of self-management, which is explained as being used for chronic illness [15]. The World Health Organisation has not defined a time aspect for when the concept of self-care can be used [16]. Its definition of self-care is more comprehensive: “The ability of individuals, families and communities to promote health, prevent disease, maintain health, and cope with illness and disability with or without the support of a health-care provider.” The same applies for the definition from the National Board of Health and Welfare in Sweden, which defines self-care as “when a patient performs health care activities at home, either by himself/herself or with the help of, for example, a relative or a personal assistant” [17]. In this review, self-care is defined as taking responsibility for one’s own preparation before surgery and for recovery after surgery to achieve well-being and health during the perioperative period.

Preoperatively, patients are expected to prepare themselves for surgery, whereas the postoperative process aims at regaining the activities of daily life and psychological well-being that existed before surgery [18]. The rapid development of fast-track surgeries has put more emphasis on detailed information from the healthcare system, such as mapping risks for complications, readmissions, and economic consequences. Furthermore, suggestions for the collection of patient-reported outcome measures are growing [19]. If e-health applications should not only be a further place for information retrieval but also support patients to take on the responsibility for their postoperative care, knowledge about how to promote self-care in this context is of importance motivation this review. The population selected for this review includes both patients and providers of perioperative care since the use of e-health applications affects both perspectives. The aim of this review is therefore to give an overview of e-health applications designed for self-care associated with surgery by collecting perspectives from both providers and patients. As our research question is broad and has the purpose of capturing the breadth of previously acquired knowledge, the methodology of a scoping review was chosen. This in line with Peters et al., (2020), who describe the methodology used in a scoping review appropriate when the research question is broad as well as key factors within a specific area are sought. We posed the following questions to the research literature:

- ❖What kind of willingness to engage in e-health is identified?
- ❖ How is motivation and supportive self-care created in perioperative e-health applications?
- ❖ Which behaviour changes are identified?
- ❖ What is the adherence to self-care information?
- ❖ What are the effects on the path of recovery (time, symptom management, complications, hospital visits, and readmission)?
- ❖ Which age groups can benefit from perioperative e-health applications?
- ❖ Which surgical procedures are studied?

## Methods

This review follow the framework outlined by Arksey and O’Malley in 2005, and further developed by Peters et al., (2020) members of Joanna Briggs Institute. The original framework consisted of five stages, that is; identify the research question; identifying relevant studies; study selection; charting the data; collating, summarising and reporting the results [20]. The authors also gave an optional sixth stage; consultation exercise, which is not employed in this study. A protocol for the review was registered with the Open Science Framework on 13 August 2020 (https://doi.org/10.17605/OSF.IO/R3QND). The protocol was informed by the Joanna Briggs Institute manual for scoping reviews [21], and the results are reported according to the PRISMA guidelines [22]. The search terms (Table 1) were chosen in collaboration with a research librarian (E.N.), who also conducted the searches.

**Table 1:** Search terms

Telemedicine/nursing	Patient outcome assessment	Participation
E-health	Patient reported outcome measures	Self-care
M-health	Self-reported assessment	Self-efficacy
Medical informatics	Self-monitoring	Self-management
Electronic mail	Communication	Empowerment
Text messaging	Decision-making	The operative period
Mobile applications	Education	Preoperative
Recovery	Surgeries procedures	Postoperative

The following inclusion criteria were used: a) e-health (smartphone, tablet device, computer) applications designed for self-care for any type of surgery involving a skin incision, b) providers of perioperative care incorporating described e-health applications, c) adult in- and out-patients (≥ 18 years) who have undergone any type of surgery with a skin incision and had access to a surgical e-health application, d) interventions comparing e-health applications with standard care, e) qualitatively analysed experiences of providers and/or patients, f) phenomenon of interest was the impact of the use of the e-health application on patients’ self-care and their recovery, and g) outcomes describe what stimulates and motivates patient self-care. In this review, e-health applications are defined as smartphone/computer tablet applications designed for providing information and communication regarding acute care, that is, perioperative information and self-assessed progression of recovery to support self-care. Surgery is defined as a procedure in which an incision is made in the skin. The following exclusion criteria were used: a) e-health applications only focusing on healthcare providers’ interaction, b) sources older than 5 years, c) adolescents and children, and d) reviews. The reasons for limitation was that the rapid digital development may have influenced the design of e-health applications and the last reason was that patients' use of applications may have increased, influencing their willingness and motivation [23]. Publications were limited to peer-reviewed articles in English, Danish, Norwegian, and Swedish from 1 January 2015 to the date of the final search on 2 June 2020.

Literature searches were developed by a research librarian (E.N.) to reflect the concepts outlined above, using controlled vocabulary whenever possible and supplemented with keyword searches in the title and abstract fields. Searches were run in the following subject databases known for indexing publications that would probably be most relevant to the research question: CINAHL with Full Text (EBSCOhost), MEDLINE (EBSCOhost), and PsycInfo (ProQuest). The search strategy was further expanded to include citation platforms that could identify additional articles of interest: Scopus (Elsevier) and Web of Science Core Collection (SCI-EXPANDED, SSCI, A&HCI, CPCI-S, CPCI-SSH, ESCI). In addition, the internet was searched through Google Scholar (Publish or Perish Software). See Additional file 1 for full documentation of the search strategies.

Records found during the search phase were exported to reference management software (EndNote) to enable the identification and removal of duplicates [24]. Prior to the formal screening process, a calibration exercise was undertaken to pilot and refine the screening questions. Records were then screened using Rayyan QCRI, a web-based application for systematic reviews [25]. Selection was based on the previously described exclusion/inclusion criteria. The sample was divided into three parts, and each part was reviewed by two members who independently performed the screening process on the title/abstract level as well as conducted the full-text assessment of included records. Any disagreements during the screening process were resolved by discussion and consensus between the three reviewers (LW, KS, KE).

**Fig. 1:**
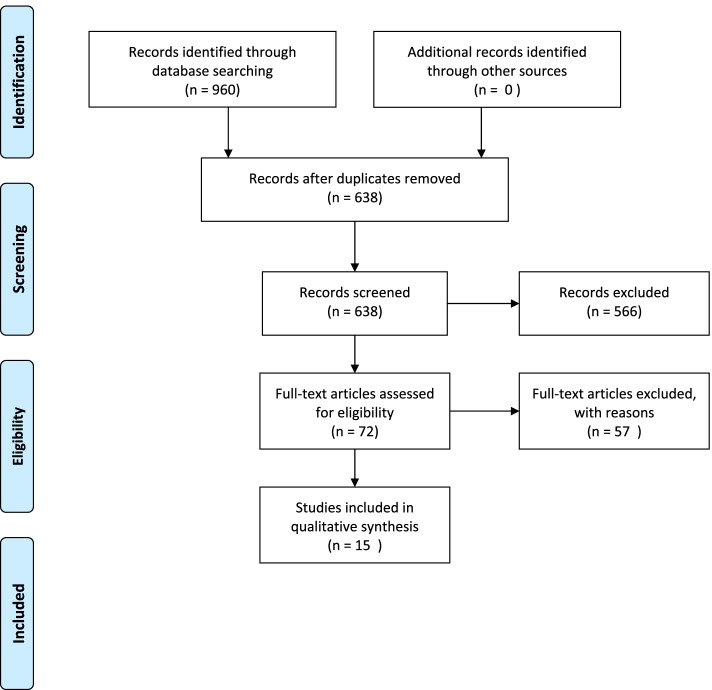
The selection process

Data charting was undertaken in accordance with our predefined research questions. The data from the results sections were searched by the three reviewers (LW, KS, KE) and discussed until consensus was achieved, as recommended in the Joanna Briggs manual [21]. Studies that did not present data in accordance with our research questions were excluded at this stage (Fig. 1).

In addition to the results, the following data from the included studies were collected and tabulated as outlined by Evans [26]: author, publication year, country, characteristics of the study population, objectives of the study, study design and methods, and intervention content and characteristics (Tables 2 and 3).

**Table 2:** Characteristics of studies included in scoping review

Authors and Country of origin	Sample (%)	Age	Setting	Intervention characteristics
**Abelson et al. 2017 **[27] USA	Individuals: *n* = 739 Intervention/control group: n/a Gender: female 368 (50)	18–34: 200 35–49: 201 50–64: 213 ≥ 64: 125	General population	Survey questions concerning the public’s access to and willingness to use mobile health technologies after surgery
**Bouwsma et al. 2018 **[28] Netherlands	Individuals: *n* = 433 Intervention/control group: 227/206 Gender: n/a	M: 46(± 7)/46(± 7)	Gynaecology	An interactive web portal facilitated self-management through the surgical pathway, providing individual tailored convalescence advice preoperatively. The web portal contained an interactive self-assessment tool to monitor recovery. Patients were advised to resume their work activities gradually to reach full return to work within a predefined number of weeks
**Cnossen et al. 2016 **[29] Netherlands	Individuals: *n* = 38 Intervention/control group: n/a Gender: female 9 (24)	M: 65, range 46–78	Laryngectomy	An app delivered a self-care education program and an exercise programme. Education and self-care management provided general information about the larynx, laryngeal cancer, and functional changes after total laryngectomy. The themes of self-care information were nutrition, tracheostomy care, voice prothesis care, speech and smell rehabilitation, mobility of head, neck, and shoulder muscles, which were illustrated with video animations, images, photos, and video demonstrations
**Davidovitch et al. 2018 **[30] USA	Individuals: *n* = 268 Intervention only/intervention and ordinary care: 99/169 Gender: female 62(63)/94 (56)	61(± 10)/66(± 10)	Total hip arthroplasty	A customizable electronic app specifically designed for rehabilitation after hip arthroplasty contained surgeon-specific videos to prepare patients before surgery and videos focusing on wound management and rehabilitation. Rehabilitation metrics offered patient activity together with the possibility of sharing images with their care teams
**Felbaum et al. 2018 **[31] USA	Individuals: *n* = 56 Intervention/control group: n/a Gender: female 33 (59)	M: 52 (± 14)	Neurosurgery	An app asked the patients to perform specific tasks, such as reading instructions. Confirmation of completed tasks was sent to the web portal, to which staff had full access. Patients also received specific timely reminder text messages. Additionally, patients could send their pain-scores and wound images to staff, enabling communication
**Glauser et al. 2019 **[32] USA	Individuals: *n* = 30 Intervention/control group: n/a Users/nonusers: 8/22 Gender: female 5(62)/10(45)	M: 51/55	Spine surgery	An app included preparation for surgery, preoperative risk mitigation, activity monitoring, wound care, and opioid use management, providing real time viewing of wound healing, activity and pain levels, and communication with providers. Patients were given a “daily to-do list” with video instructions and given a star when competed. The “to-do list” included activity levels and diet, as well as possibilities for reporting symptoms daily. Individual trends were shown graphically for both patients and providers
**Gustavell et al. 2019 **[33] Sweden	Individuals: *n* = 59 Intervention/control group: 26/33 Gender: female 9(35)/13(39)	M (SD): 67(9)/66(9)	Pancreaticoduodenectomy	An app in which daily regular patient reports of self-assessed symptoms were requested. A reminder was sent every day. Patients was offered continuous access to evidence-based self-care advice, and graphs to view their history of symptom reporting. In the case of an alert, patients were contacted by their contact nurse
**Hou et al. 2019 **[34] China	Individuals: *n* = 168 Intervention/control group: 84(50)/84(50) Gender: female 57(48)/50(42)	M (SD): 51(10)/49(10)	Spine surgery	The app contained two interfaces: a mobile-phone-based interface for patients and a web-based interface for doctors. Patients could view their rehabilitation plans that included individual video instructions. Patients received daily exercise reports and alerts to prompt them to return to the system. They could communicate with their doctors, who continuously adjusted rehabilitation plans
**van der Meij et al. 2018 **[35] Netherlands	Individuals: (*n* = 344) Intervention/control group: 173(50)/171(50) Gender: female 95(55)/92(54)	M: 52, range42-61/51, range41-58	Gynaecological and abdominal general surgery	The intervention care program comprised a website, an app, and an activity tracker, preparing patients for surgery and supporting them postoperatively. Patients could develop a personalized convalescence plan, access information about the perioperative period by text and video animation, use and monitor personal feedback on the recovery process, and had use of an e-consult function
**Mundi et al. 2015 **[36] USA	Individuals: *n* = 30 Intervention/control group: n/a Gender: female 27(90)	M (SD): 41(11)	Bariatric surgery	The app contained components aimed at educating, assessing, and engaging patients, that is, brief text messages encompassing lifestyle domains and short video-based education modules, which were followed to verify mastery of the topic. Tailored messages were electronically generated and sent to patients to modulate behaviour. Patients received either a congratulatory or supportive messages
**Pecorelli et al. 2018 **[37] USA	Individuals: *n* = 45 Intervention/control group: n/a Gender: female 16(36)	≤ 50 11(24%) 50–70 22(49%) > 70 29(27%)	Bowel surgery	An app included patient education, reminders of daily recovery milestones, and questionnaires to track patients’ adherence to the recovery process and assess patient-reported outcomes. The app provided feedback on adherence to individual recovery elements and encouraged to reach daily goals. Motivation was enhanced by letting patients do private internet browsing and messaging on the iPad
**Pickens et al. 2019 **[38] USA	Individuals: *n* = 122 Intervention/control group: n/a Gender: n/a	n/a	Hepatopancreatobiliary surgery	A web-based platform accessible by any smartphone and tablet device was customized to an ERAS programme. Patients were provided with scheduled task reminders for preoperative preparation and prompted to access a digital education library reviewing details of their medical condition, scheduled operation, and anticipated ERAS expectations. From the day of surgery, the app provided prompts to complete a daily survey of symptoms, opiate use, anxiety, and quality-of-life scores. Responses triggered guidance for self-care at home, call to a nurse, or to seek immediate attention at the Emergency Department. Patients were encouraged to involve their family and friends
**Russ et al. 2020 **[39] England	Individuals: *n* = 42 Intervention/control group: n/a Gender: female 25(59)	18–34 17(40%) 35–64 21(50%) ≥ 65 4(10%)	General-, orthopaedics, obstetrics, eye-, gynaecological and other surgery	An app aimed to enhance safety in the surgical process and provide evidence-based simple information and animations around specific areas of risk for safety: preparing for surgery, personal details, consent, hand hygiene, deep-vein thrombosis, falls, pressure ulcers, medications, wound care, nutrition, and going home. The app provided step-by-step advice on the actions that patients and their intimates could take, including warning signs to look out for, information, and questions to ask
**Timmers et al. 2019 **[40] Netherlands	Individuals: *n* = 213 Intervention/control group: 114(54)/99(46) Gender: female 74(65)/60(61)	M (SD): 65(8)/66(8)	Knee replacement	The app offered day-to-day information. Push notifications were used to actively alert patients about information that was available. The text, photos, and videos in the intervention were based on existing protocols. After discharge, patients received supporting information on pain, physiotherapy exercises, wound care, and daily self-care activities. Additionally, patients were requested to enter their pain scores and were able to view their results in a graph. They could also upload a photo of their wound
**Tofte et al. 2020 **[41] USA	Individuals: *n* = 16 Intervention/control group: n/a Gender: female 11(69)	M: 48, range: 23–63	Carpal tunnel release	The app included online modules corresponding to elements of a typical postoperative visit: dressing removal, suture removal, documentation of wound appearance, evaluation of nerve symptoms, and documentation of a physical exam, including motor exam and range of motor. Video instructions guided patients through dressing removal and suture removal. Patients then uploaded a wound photo, completed a self-assessment of median nerve symptoms, and recorded a video of physical exam manoeuvres

**Table 3:** Objectives, study design, methods and results in studies included in the scoping review

Study Country of origin	Objective	Study design	Results
**Abelson et al. 2017 **[27] USA	To determine NY State residents’ willingness to engage in mHealth after surgery and compare socioeconomic factors that may affect willingness to engage	Survey	Primary outcome: The majority reported a willingness to engage with mHealth, including wearing a tracker on their wrist, filling out daily surveys, sending pictures, and sharing updates Secondary outcome: Higher education, trust on the internet, and pre-existing smartphone use were associated with higher willingness to engage with mHealth. Black race and Hispanic ethnicity, but not age, were associated with lower willingness
**Bouwsma et al. 2018 **[28] Netherlands	To study the implementation of the care programme in the daily practice of nine hospitals in the Netherlands	Multicentre stepped-wedge cluster randomized controlled study	Primary outcome: The median duration until return to work was shorter in the intervention group than in the usual care group, 48/62 days Secondary outcomes: At the two-week follow-up, the intervention group differed significantly from the usual care group in quality of life (*p* = .0046), pain (*p* = .014), and disability (*p* = .000). These differences disappeared over time. There were no differences in functional health status, self-efficacy, or coping between the groups
**Cnossen et al. 2016 **[29] Netherlands	To investigate the feasibility of the selfcare education programme in clinical practice by assessing uptake and usage rate and user satisfaction of a programme supplementary to regular care The secondary aim was to investigate which sociodemographic and clinical factors are associated with user satisfaction	Multicentre single group cross-sectional study design	Usage: Sixty-nine percent of the patients logged in, 55% spent < 60 min. using the programme, 29% 60–90 min. and 16% > 90 min. in the study period of 2 weeks. The majority (84%) found the programme beneficial in managing self-care and reported no problems performing self-care. Three patients had technical problems watching videos, and three were not interested
**Davidovitch et al. 2018 **[30] USA	The aim was to investigate the clinical utility and economic burden associated with digital rehabilitation apps in primary total hip arthroplasty (THA) recipients	Single-centre retrospective review	Both the platform-only and the platform-HHS cohorts demonstrated similar improvements in all PROs at 12 weeks. The platform-only cohort was significantly (*p* = < 05) more likely to download the mobile platform and demonstrated a significantly (*p* = .0001) greater engagement with the platform
**Felbaum et al. 2018 **[31] USA	To demonstrate that app-based instructions with built-in reminders may improve patient understanding and compliance and contribute to reducing the number of surgery cancellations and postoperative complications and readmissions	Prospective evaluation	Fifty-four (96%) of the 56 included patients successfully downloaded, registered and used the studied app There were no cancelled surgeries, and one postoperative complication was registered. Eight patients called the office on a single occasion regarding perioperative care
**Glauser et al. 2019 **[32]	The objective of this innovative app is to integrate enhanced recovery after surgery (ERAS) principles, patient education, and real-time pain and activity monitoring in a home setting with unencumbered two-way communication	A quality improvement effort	Eight (27%) of 30 patients logged in nearly every day from a week pre-op to > 45 days post-op. They found the daily reminders and graphical presentations of their trends most helpful. Helpful was also the daily to-do list, wound pictures, walking measures, pain level tracking, and communication with care. The non-users (*n* = 22, 73%) chose not to use the app due to no interest, difficulties with registration, difficulties using it, and not remembering to use it
**Gustavell et al. 2019** [33] Sweden	The aim was to evaluate the impact on health-related quality-of-life and self-care activity when using the Interaktor app following pancreaticoduodenectomy due to cancer	Historically controlled single-centre design	Health-related quality-of-life ratings at 6 weeks and 6 months were significantly higher in the intervention group. At 6 months, the intervention group rated higher (*p* = .033) engagement in self-care activities. The intervention group reported non-significant changes in 21 of health- related quality of life as compared to 8 for the control group In the first 4 weeks, the intervention group reported symptoms as intended, (Mdn) 95%. Alerts were triggered (Mdn) three times. Self-care advice was viewed (Mdn) 13.5 times, mostly regarding pancreatic enzyme supplements, dietary advice, and pain From 4 weeks to 6 months, the adherence was (Mdn) 83%. Alerts were triggered (Mdn) 7 times. Self-care advice was viewed (Mdn) 11 times
**Hou et al. 2019 **[34] China	The aim of this study was to examine the efficacy of mobile-phone-based rehabilitation systems in patients who underwent lumbar spinal surgery	Multicentre prospective RCT	A total of 50 (78%), 37/58%), and 38 (59%) patients had high compliance at 6, 12, and 24 months, respectively. Twenty-four (37%) completed the whole trial. Reasons for low compliance included lack of communication with doctors, concerns about the accuracy of the action, limited symptom improvement, and lack of motivation Primary outcomes: The Owestry Disability Index (ODI) and pain were high in both groups, and no significant differences were seen at baseline up to 12 months. At 24 months, ODI and pain were significantly (*p* = < .05) improved in the e-health group Secondary outcomes: No significant differences between groups were found for movement at 3, 6, and 12 months; at 24 months, there was a significant (*p* = < .05) improvement in the e-health group. The EuroQol-5 improved significantly (*p* = < .05) in the e-health group at 6, 12, and 24 months. The SF-36 improvement was significant (*p* = < .05) at 3, 6, and 24 months in the e-health group. In the group with the highest adherence (subgroup with e-Health), there were significant (*p* = < .05) changes in ODI and pain at 6, 12, and 24 months
**van der Meij et al. 2018 **[35] Netherlands	The effect of a personalized e-healthcare programme on return to normal activities after surgery was evaluated	A multicentre, single-blind, randomized controlled trial	100% completed the baseline questionnaire, and questionnaires at 1, 3, and 6 weeks, and 3 and 6 months after surgery were completed by, respectively, (95%), (94%), (90%), (88%), and (87%) Primary outcome: Median time until return to normal activities was significantly (*p* = .007) shorter in the intervention group Secondary outcomes: Median time until 75% of normal activities and time until full resumption of work were in favour of the intervention group. Up to 6 months social participation (*p* = .038) and physical function (*p* = .024) scores were significantly higher in the intervention group; other measures did not differ between groups
**Mundi et al. 2015 **[36]	The primary objective was to assess the feasibility of using a smartphone app with EMA/EMI functionality to prepare patients for bariatric surgery	Ecological Momentary Assessment (EMA)/intervention study	Twenty (67%) out of 30 patients pursued the study. On average in this group, seven out of nine app modules were completed. There was a correlation between EMA response and confidence in maintaining an exercise regimen. The app was reported as being helpful in preparing for surgery. A small increase in nutrition knowledge and feeling more engaged in healthy lifestyle was seen. Two (10%) of twenty patients did not pursue surgery due to weight loss
**Pecorelli et al. 2018 **[37]	The objective was to assess the validity and usability of a novel mobile device app for education and self-reporting of adherence for patients undergoing bowel surgery within an established ERP	A prospective, single-group pilot study follow-up	Patients used the app a median of 10 min/day. Patients completed 89% of available questionnaires. Reasons for non-completion were ongoing postoperative complications (*n* = 8), patient forgot (*n* = 7), patient did not understand the task (*n* = 2), and technical issues (*n* = 2). The majority (89%) of patients found the app to be very helpful in understanding and achieving recovery goals and reported increased motivation to recover after surgery
**Pickens et al. 2019 **[38] USA	The aim was to demonstrate the novel implementation of an established mobile health app for PRO collection in an ERAS programme for hepatopancreatobiliary (HPB) surgery	A prospective, single-group pilot study	Patient engagement was 93% (114/122) before surgery. Immediate postoperative engagement was 88%, after discharge 52%, and 30% at week 3. Patients submitted 57% of prompted PROs. The 30-day end-of-study PROMIS survey was completed by 41% The app helped the majority (29/30, 97%) feel more prepared before surgery and allowed (69/88, 78% to “feel more confident” and “worry less” during their recovery. Twenty-five to 36 patients reported compliance with self-care day 1–7. Twelve patients reported that the app prevented phone calls to the office and a visit to the ED. Most alerts (393/521) needed no further help due to given guided responses to further self-care or to call the nurse. Seven patients were directed to an ED
**Russ et al. 2020 **[39]	The research objectives were twofold: 1. To assess the views relating to the app with a cohort of diverse surgical patients recruited from the community and to understand perceptions of the app, perceived impacts on care and safety, and areas of improvement 2. To describe and evaluate the approach and impact of incorporating diverse PPI into the project design, planning, and delivery	Participatory action research	There were no significant differences in perceptions of the app according to sex, age, ethnicity, or length of hospital stay. Those with a disability were significantly less likely to agree that the app was easy to use. Those who had experienced previous surgery were significantly more positive. The majority experienced help in conversations around their care and changed the way they behaved. One third encountered technical difficulties when downloading the app Reflective notes: Patients could care better for themselves; the app promoted confidence and a sense of security, reduced worry, and made them feel less alone
**Timmers et al **[40] **2019**	The aim was to investigate the effect of an interactive app on patients’ level of pain, physical functioning, quality of life, satisfaction, and healthcare consumption in the first four weeks of recovery after total knee replacement (TKR)	Randomized controlled trial	Primary outcome: In the intervention group, pain was significantly lower during rest, activity, and night at postoperative weeks 2, 3, and 4 Secondary outcomes: Physical functioning (*p* = . < 001) and quality of life (*p* = . < 001) were significantly better in the intervention group at 4 weeks postoperatively. In both groups, the ability to perform physiotherapy exercises and daily self-care activities during the first 4 weeks after discharge increased, and the intervention group performed better from the second week onwards. In both groups, perceptions of hospital involvement in their recovery process decreased during the 4 weeks; the intervention group had a smaller decrease. There was a significant (*p* = .014) difference in health consumption, in favour of the intervention group. The app was primarily used the first 2 weeks. Information and videos on pain, wound care, physiotherapy exercises, and self-care activities were most frequently used
**Tofte et al. 2020 **[41]	The purpose was to (1) implement a software platform to remotely and asynchronously accomplish the typical requisite elements of a postoperative CTR visit, (2) determine whether patients can reliably accomplish these tasks without direct supervision in clinic, and (3) describe characteristics of the software interface and patient population that were associated with patient success	Intervention prospective cohort study	Twelve of 16 (75%) of patients underwent surgery on their dominant extremity. The average time to complete the software modules was 13 min. Eleven (69%) patients used help from an intimate to complete the study objectives. All patients successfully completed dressing removal. Ten (62%) removed their sutures. Fourteen (88%) captured wound photos and classified the wound status. Fifteen (94%) patients successfully answered a question about median nerve symptoms, and 14 (88%) captured a physical exam video. Eight (50%) completed all aspects of the software module. No significant wound complications were identified. Hand dominance, age, self-perception of tech savviness, volume of text messaging, surgical technique, and phone usage activities were not predictive of successful study completion

In the synthesis of results, all results corresponding to the research questions in the included studies were compiled by the main author (LW). Identified data were categorized for each research question by the main author and discussed by the three reviewers (LW, KS, KE) to ensure consensus. The quantitative measures were presented with descriptive statistics, and the presentation of the qualitative synthesis followed the steps of Braun and Clarke for theoretical (deductive) thematic descriptive analysis [42].

## Results

A total of 15 studies were identified for inclusion in the review. The searches provided a total of 960 citations. After adjusting for duplicates, 638 remained. Of these, 566 studies were discarded because, after reviewing the abstracts, these papers clearly did not meet the criteria. The full text of each of the remaining 72 citations was examined in more detail, and 57 studies did not meet the inclusion criteria as described. No unpublished relevant studies were obtained. See flow diagram Fig. 1.

### Study characteristics

The 15 reviewed studies were conducted in Asia (China), Europe (England, Netherlands, and Sweden), and North America (Canada, USA) and in several surgical contexts (abdominal, eye, gynaecological, orthopaedic, and throat). Participants’ ages ranged from 18 to 78, and the gender distribution in the study populations varied: 24–90% were women. The length of programmes varied between 2 weeks and 24 months. Five of the studies were randomized controlled trials [28, 33–35, 40], eight were cohort studies [27, 29–31, 36–38, 41], and two had a mixed-method approach [32, 39]. No study explored the providers’ perspectives. Recommended self-care actions in the studied applications were not clearly described in all studies. Table 4 describes the identified preoperative and postoperative self-care actions included in the studied applications.

**Table 4:** Identified self-care actions

Identified self-care actions	Studies
Preoperative:	
• life-style changes preparations • preparations	• Hou et al. 2019 [34], van der Meij et al. 2018 [35], Mundi et al. 2015 [36], Timmers et al. 2019 [40] • Bouwsma et al. 2017, Felbaum et al. 2018 [31], Glauser et al. 2017, van der Meij et al. 2018 [35], Mundi et al. 2015 [36], Pickens et al. 2019 [38], Russ et al. 2020 [39]
Postoperative:	
• activity/exercises • management of surgical wounds/tracheostomy/removing of sutures • nutritious food • pain management • self-assessments of postoperative recovery (activities, exercises, and symptoms)	• Cnossen et al. 2016 [29], Davidovitch et al. 2018 [30] • Cnossen et al. 2016 [29], Davidovitch et al. 2018 [30], Russ et al. 2020 [39], Tofte et al. 2020 [41] • Cnossen et al. 2016 [29], Mundi et al. 2015 [36], Russ et al. 2020 [39] • Glauser et al. 2017 • Bouwsma et al. 2017, Glauser et al. 2019 [32], Gustavell et al. 2019 [33], Pecorelli et al. 2018 [37], Pickens et al. 2019 [38], Timmers et al. 2019 [40], Tofte et al. 2020 [41]

### Identified willingness to engage in e-health

One study examining patient willingness reported potential users of surgical e-health to be interested and willing to engage. However, the researchers concluded that some ethnicities may need education regarding e-health technology before choosing this alternative for self-care support [27]. Two other studies reported that self-perception of tech savviness [32, 41], experiences of text messaging, and smartphone usage [41] were not necessary prerequisites for willingness to complete tasks asked for in the e-health applications. However, other barriers to engagement in e-health applications were identified. These were technical issues, such as problems with downloading and registering the applications and starting videos [29, 32, 35, 37]. In addition, the use of the e-health applications was also affected by ongoing postoperative complications [35, 37], forgetfulness [32, 37], or not understanding the task [37].

### Motivation and supportive self-care created in surgical e-health applications

How motivation to self-care is created in e-health applications was not the primary focus of any of the studies. However, in many of the included studied e-health applications, supportive functions such as information, messages, alerts, and opportunities to get in touch with healthcare professionals could be interpreted as motivational for performance of self-care (Table 5).

**Table 5:** Identified motivational factors to self-care in pre and postoperative E-health applications

Identified motivational factors to self-care	Studies
Private use of borrowed tablets	Pecorelli et al. 2018 [37]
Chat features	Davidovitch et al. 2018 [30], Felbaum et al. 2018 [31], Glauser et al. 2019 [32], Gustavell et al. 2019 [33], Hou et al. 2019 [34], Pickens et al. 2019 [38]
Encouragement of involvement with family and friends	Pickens et al. 2019 [38]
Educational/instructive texts, photos and/or videos	All included studies
Monitoring of self-assessed recovery and shown in graphs	Bouwsma et al. 2017, Glauser et al. 2019 [32], Gustavell et al. 2019 [33], Pecorelli et al. 2018 [37], Timmers et al. 2019 [40]
Personalized care plans	Hou et al. 2019 [34], van Der Meij et al. 2018 [35]
Sharing of, • Photos • videos	• Davidovitch et al. 2018 [30], Felbaum et al. 2018 [31], Glauser et al. 2017, Timmers et al. 2019 [40], Tofte et al. 2020 [41] • Tofte et al. 2020 [41]
Reminders and messages	Felbaum et al. 2018 [31], Glauser et al. 2019 [32], Gustavell et al. 2019 [33], Hou et al. 2019 [34], Mundi et al. 2015 [36], Pecorelli et al. 2018 [37], Pickens et al. 2019 [38], Timmers et al. 2019 [40]
Alerts	Hou et al. 2019 [34], Gustavell et al. 2019 [33]

In all, information in the e-health applications consisted of education, instructions, or advice, with or without videos, and was meant to increase patients’ knowledge and thereby increase their engagement in self-care. Information included in the applications was about topics such as disease and surgery, preoperative preparations, and postoperative recovery. Self-care information mainly concerned preoperative preparation activities, recommended postoperative activities, nutrition, wound care, and pain management. Eight e-health applications used scheduled push notifications of daily targets with a predetermined regularity and with the intention of helping patients remember their self-care activities*.* Alerts were used in two studies to identify abnormal postoperative self-assessed reported symptoms that needed to be assessed by the care team. Six studies explained that they used evidence-based information [30, 32, 33, 37–39].

Different technical constructions in the e-health applications offered opportunities to communicate about self-care issues and symptom experiences with members of the care teams. Chat features for communication enabled written or verbal access to members in the care team and allowed for visual communication through technical possibilities such as sharing images and videos (e.g. wound pictures, rehabilitation videos). Examples of additional indirect motivating strategies included offering personalized care plans**,** tools for monitoring self-assessed recovery for reporting to the care team, and enabling patients to view their history of symptoms reported in graphs. Supportive reminders and messages, encouraging the involvement of family and friends, and allowing patients to use borrowed tablets for private issues, such as internet browsing and messaging, were other identified motivational factors. When supported by e-health, patients reported increased motivation to recover after surgery [37] and feeling more prepared before surgery, as well as feeling confident and less worried during their recovery at home [38].

### Identified behaviour changes

The results indicate that e-health applications in postoperative recovery provide increased participation in self-care. Measured self-care activities showed in one study a significant increase compared to standard care after 6 months [33] and in another study behaviour changes before surgery, could lead to the prevention of (e.g. bariatric) surgery [36]. Furthermore, two studies (pancreas and joint surgery) indicate that patients’ increasing ability to maintain physiotherapy and self-care activities over a longer period give positive effects on emotional functioning [33], physical functioning [40], quality of life, and decreased healthcare consumption [40]. One study found that properties such as functional health status, self-efficacy, or coping did not influence behaviour change [28].

### Adherence to self-care information

Among the studies where adherence was measured, only three measured self-care activities [34, 36, 41]. In Hou et al., adherence to training sessions was reported to be higher with e-health than with standard care [34].

Instead of measuring self-care activities, various technical measures of how patients used the e-health applications showed a great variation, from 27% up to 100%, between the studies [29–35, 37, 38, 40]. Four studies presented results on adherence using the e-health application over time and showed that adherence was not maintained but decreased from 93% before surgery to 30% after surgery [38], from 95 to 85% in 6 months [33], from 100 to 94% in 6 months [35], and from 100%-63% in 24 months [34]. Reasons for low adherence to e-health applications included experiences of lack of communication with the care team, accuracy of described self-care actions, limited symptom improvement [34], and lack of interest [29, 32, 34, 35].

### Effects on the path of recovery (time, quality of life, functioning, symptoms, consumption of healthcare)

The impact of e-health applications on recovery time was shown in two studies where the intervention groups returned to work (Mdn 49 days, intervention group/62 days, usual care group) or normal activities (Mdn 21 days intervention group/26 days usual care group) earlier than the groups with standard care [28, 35]. Self-care activities regarding pain were not directly measured in most studies; however, the presence of pre- and postoperative symptoms and recovery and quality-of-life measures can be assumed to reflect self-care activities. The results in Mundi et al. (2015) showed that e-health applications with self-care advice can improve symptoms i.e. weight loss before surgery. Furthermore, results from five of the included studies displayed positive impact on the path of recovery. Postoperative symptoms [28], quality of life [28, 33] and disability [28] differed positively from usual care in the short (2–6 weeks) postoperative perspective. In the longer (3–24 months) postoperative perspective positive impact on quality of life [33–35], postoperative symptoms [30, 34] and physical functioning [34, 35] was still shown.

In two studies patients’ consumption of healthcare, that is, telephone calls and physical visits, decreased due to the supportive effects of the surgical self-care applications [38, 40]. None of the studies presented results on whether the applications affected the number of postoperative complications and readmissions.

### Groups that can benefit from pre- and postoperative e-health applications

Concerning this research question, no study was found; therefore, the mean age and range of included participants are described (see study characteristics), which indirectly reflect that people of a wide variety of ages have engaged in surgical self-care supported by e-health applications.

## Discussion

E-health applications have become increasingly common in many countries over the last several years [43]. Sweden belongs to the countries that have the highest level of e-health adoption [44]. Our study aimed to give an overview of e-health applications designed for self-care associated with surgery from the perspectives of providers and patients.

The main findings revealed that the most common self-care actions included preoperative preparations [28, 31, 32, 35, 36, 38, 39] and self-assessments of postoperative recovery [28, 32, 33, 37, 38, 40, 41], while the most commonly identified motivational factors for self-care in pre- and postoperative e-health applications included supportive reminders and messages [31–34, 36–38, 40] and chat features [30–34, 38]. There was a great variance in research design and technical solutions. In spite of this, patients’ willingness to engage with and adherence to the different e-health applications seemed to increase self-care activities [33, 36, 40] and thereby accelerated return to work and normal activities [28, 35], resulting in an apparent decrease in the patients’ need for physical healthcare visits [38, 40]. Despite the fact that age groups were not primarily explored, the included studies showed that adult patients of any age engaged in surgical self-care supported by e-health. No studies were found that explored providers’ perspectives.

### Motivation and supportive self-care created in surgical e-health applications

None of the studies focused primarily on how motivation to self-care was created in e-health applications. However, from a technical point of view, the most common way to try to motivate patients seemed to be supportive reminders and messages [31–34, 36–38, 40], while sharing of videos [41] and encouragement of participation with relatives [38] were only sparsely identified. McCarron et al. found that individuals were motivated not only to satisfy their needs but also to maximize the value they received [45]. The challenge for developers of e-health supporting self-care is to create applications that most patients consider feasible.

Understanding what motivates patients to become engaged is a way to highlight what is important for them. Lancaster et al. concluded that e-health applications with multifaceted functionalities and those allowing for direct patient-provider communication may be more effective at improving patient self-care [46]. Similar results were shown by Wentink et al., who found that patients identified their e-health needs as including online access to their health record, communication with family, and the possibility of scheduling meetings with providers [47]. In six of the reviewed studies [30–34, 38], chat features with the care team were offered. Patients’ access to chat features is a relatively new channel for communication. However, meeting patients’ expectations of quick responses may constitute a challenge for a care team since patients are used to quick answers when searching for information on the internet.

### Identified behaviour changes

E-health applications in postoperative recovery seem to increase participation in self-care compared to standard care, which is in line with studies with similar topics [46]. An increased adherence to prescribed medications was found when patients were supported by e-health.

Symptom resolution is an outcome shown to be of most interest by providers. As a result of this, patients are advised not only to take their medication but also to change their activity level, modify their diet, or call the provider [48]. We found in our review that self-care activities, that is, behaviour changes, were mostly measured through information based on how well the form was filled in, for example, if predefined activities were performed, whether symptoms were present, and how long it took for patients to return to full-time work.

Factors other than those previously mentioned may be of more importance in affecting change in patient behaviour. Osokpo and Riegel found that culture, specifically in African American and South Asian populations, influenced self-care in general but particularly influenced the maintenance of self-care behaviours [49]. This is in line with Hou et al., who found that Chinese patients wanted support from the care teams [34]. Authorities, such as healthcare professionals in care teams, may shape a more passive and submissive attitude that affects the ways patients interpret and report their self-care and behaviour changes [50]. According to Jönsson et al., it is important that caregivers recognize their prejudices and assumptions about individuals who are different [51].

### Adherence to self-care information

In four of the 15 included studies, patients’ adherence to using and registering their recovery in the e-health applications gradually decreased over time. All these studies indicated that lack of interest was the reason why patients stopped using the application. One reason for the lack of interest might be that once patients started to feel recovered, support from the application was no longer needed. However, one of the studies in our review presented alternative reasons for discontinuing the use of applications [34]. In this study, patients had doubts during their rehabilitation, such as whether the actions were standard, whether the intended goal of rehabilitation was appropriate, or if more motivation was needed. This is in line with a study about adherence to self-care that found a number of different reasons that, in summary, described patients’ lack of understanding of the significance of continued self-care [52]. Therefore, when creating self-care applications, it is important to take advantage of the knowledge that already exists and to continue research aimed at understanding patients’ reasoning when choosing and maintaining self-care activities. Furthermore, standard care needs to be retained as an option for those patients for whom the use of an application is not suitable or feasible.

### Effects on the path of recovery (time, symptom management, complications, hospital visits and readmission)

Our study shows that patients using e-health applications were found to return to work earlier than patients who received standard care [28, 35]. These results suggest that there may also be socio-economic incentives linked to increased use of programmes in e-health applications, not only from the patients’ perspective but also from a socio-economic perspective, including healthcare insurance and healthcare in general. Wentink et al. found that long-term medical care needs could be addressed at significantly lower expenditures with e-health solutions by means of improved accessibility to rehabilitation programmes for patients with mobility impairments [47]. Further research is needed to evaluate the socio-economic effects of supporting patients’ surgical self-care by using e-health.

### Groups that can benefit from pre- and postoperative e-health applications

We found no study investigating which patient groups may benefit from pre- and postoperative e-health applications. In a review by Jonker et al., elderly surgical patients considered e-health interventions usable, satisfying, and acceptable [53]. This is in line with our included studies in which several elderly people were engaged. Other patient groups that have been mentioned as benefitting from e-health are those in need of rehabilitation [47]. However, Scherer et al. highlighted that even if most patients saw a direct benefit for themselves with e-health applications, there were also patients who were concerned about data protection. In conclusion, when care teams offer e-health applications collecting personal health-related information, data security is of great importance [54].

### Study limitations and strengths

We have aimed to give an overview of e-health applications designed for self-care associated with surgery. According to the chosen design of the review [21], we decided not to review the quality of the included studies; however, all the included studies were peer reviewed, and one third were also randomized controlled trials, which attributes a certain quality to the results.

To achieve trustworthiness, the search strategy was performed together with a university librarian, which provided quality for keyword selection and search strategies. To ensure dependability, the whole search process was described step by step, which shows a striving for methodological rigour [21]. Due to the wide variation in study design, profound discussions by three of the authors who have knowledge of both quantitative and qualitative methods, surgical care processes, self-care, and e-health were necessary to achieve consensus regarding the final inclusion of studies. Also, to increase credibility, each article was reviewed by two members (K.E. + L.W., K.E. + K.S., or L.W. + K.S.), who independently performed all steps in the screening process. Any disagreements in the analysis phase were discussed with the third member until consensus was reached [55].

As a descriptive synthesis means several threats to the validity of a review, a rich presentation of the included studies has been attached in two tables. This kind of tabulation allows readers to create their own perceptions of the body of research [26].

Transferability of the results must be done with the understanding that the use of e-health in surgical self-care is a new research area. The short history of e-health applications in surgical care may not only explain the great variance of self-care activities in the applications but also reflect different countries’ cultures in trusting patients’ capability to perform self-care. Another aspect is that different surgical specialities use different concepts, for example, ERAS in surgery [56], where some are validated and some are not.

## Conclusion

Despite the wide variety of surgeries and the varying level of support that e-health applications offer in surgical self-care, e-health in this context seems to have a positive impact on recovery. This means that e-health solutions supporting self-care can be a good alternative for those patients who are willing to engage in self-care and can manage the technology. However, to increase the effects of e-health, motivating technical measures to maintain patient’s adherence over time needs to be further evaluated by both providers and patients. Furthermore, e-health applications were found to include a wide variety of self-care activities, which presupposed patients’ ability to take responsibility for their care, meaning that surgical self-care activities in the home are under development. Providers and patients and their experiences of surgical self-care are valuable contributions to the development of available sustainable technological solutions at reasonable costs. Therefore, according to our findings and with a view to future care, there is a need for studies focusing on providers’ attitudes to surgical self-care at home and patients’ capabilities, needs, and wishes for accessibility and user-friendliness concerning e-health solutions.

## Supplementary Information

**Additional file 1.** Search strategies.

## Data Availability

All data generated or analysed during this study are included in this published article [and its supplementary information files: Appendix].
