# Supplementary material for: Patients’ and providers’ perspectives on e-health applications designed for self-care in association with surgery – a scoping review
Source: BMC Health Serv Res. 2022 Mar 23;22:386. doi: 10.1186/s12913-022-07718-8 (PMC8944084; doi:10.1186/s12913-022-07718-8)
Supplement: Supplementary file 1 — Additional file 1. Search strategies. [file 12913_2022_7718_MOESM1_ESM.pdf]

Appendix 1: Search strategies

All searches were run for the final time on June 2, 2020.

CINAHL with Full Text (EBSCOhost)

#	Query
S1	(MH "Perioperative Care") OR (MH "Perioperative Nursing") OR (MH "Postoperative Care") OR (MH "Postoperative Period") OR (MH "Preoperative Care") OR (MH "Preoperative Period") OR (MH "Surgical Patients")
S2	TI ((peri-operative OR perioperative OR post-operative OR postoperative OR post-surgery OR “post surgery” OR pre-operative OR preoperative OR pre-surgery OR surgery OR surgical) N3 (care OR medicine OR nurs* OR patient* OR period* OR procedure*)) OR AB ((peri-operative OR perioperative OR post-operative OR postoperative OR post-surgery OR “post surgery” OR pre-operative OR preoperative OR pre-surgery OR surgery OR surgical) N3 (care OR medicine OR nurs* OR patient* OR period* OR procedure*))
S3	S1 OR S2
S4	(MH "Telehealth") OR (MH "Telemedicine") OR (MH "Telenursing") OR (MH "Telerehabilitation") OR (MH "Mobile Applications") OR (MH "Cellular Phone") OR (MH "Computers, Hand-Held") OR (MH "Email") OR (MH "Smartphone") OR (MH "Text Messaging") OR (MH "Health Informatics") OR (MH "Medical Informatics") OR (MH "Nursing Informatics")
S5	TI ((e-health OR ehealth OR m-health OR mhealth OR tele-health OR telehealth OR tele-medicine OR telemedicine OR tele-nursing OR telenursing OR tele-rehabilitation OR telerehabilitation) OR ((cell-phone OR cellphone* OR “cellular phone*” OR “electronic mail*” OR e-mail* OR email* OR “hand-held computer*” OR “handheld computer*” OR “hand-held device*” OR “handheld device*” OR iPad* OR iPhone* OR mobile* OR “palmtop computer*” OR “palmtop device*” OR PDA* OR “personal digital assistant*” OR smart-phone* OR smartphone* OR tablet* OR SMS OR MMS OR text-messag* OR “text messag*”)N3(app* OR application* OR program*)) OR ((health OR medical OR nursing) N3 informatics)) OR AB ((e-health OR ehealth OR m-health OR mhealth OR tele-health OR telehealth OR tele-medicine OR telemedicine OR tele-nursing OR telenursing OR tele-rehabilitation OR telerehabilitation) OR ((cell-phone OR cellphone* OR “cellular phone*” OR “electronic mail*” OR e-mail* OR email* OR “hand-held computer*” OR “handheld computer*” OR “hand-held device*” OR “handheld device*” OR iPad* OR iPhone* OR mobile* OR “palmtop computer*” OR “palmtop device*” OR PDA* OR “personal digital assistant*” OR smart-phone* OR smartphone* OR tablet* OR SMS OR MMS OR text-messag* OR “text messag*”)N3(app* OR application* OR program*)) OR ((health OR medical OR nursing) N3 informatics))
S6	S4 OR S5
S7	(MH "Self Care") OR (MH "Consumer Participation") OR (MH "Decision Making, Shared") OR (MH "Empowerment") OR (MH "Patient Education") OR (MH "Patient Satisfaction") OR (MH "Self-Efficacy") OR (MH "Self-Management")
S8	TI ((self-care OR self-efficacy OR self-manage* OR self-monitor*) OR (self N3 (care OR efficacy OR manage* OR monitor*)) OR (decision-making OR “decision making”) OR (decision* N3 (make OR making)) OR empower* OR ((patient* OR client* OR consumer*) N3 (education OR participation OR satisfaction)) OR (self-reported N3 (assessment* OR outcome*)) OR recovery) OR AB ((self-care OR self-efficacy OR self-manage* OR self-

	monitor*) OR (self N3 (care OR efficacy OR manage* OR monitor*)) OR (decision-making OR “decision making”) OR (decision* N3 (make OR making)) OR empower* OR ((patient* OR client* OR consumer*) N3 (education OR participation OR satisfaction)) OR (self-reported N3 (assessment* OR outcome*)) OR recovery)
S9	S7 OR S8
S10	S3 AND S6 AND S9

Limiters - Peer Reviewed; Published Date: 20150101-20200631; Language: Danish, English, Norwegian, Swedish

GoogleScholar (Publish or Perish Software)

Keywords: surgery|perioperative|postoperative|preoperative ehealth|telehealth|telemedicine|telenursing|telerehabilitation|”mobile applications”|informatics “self care”|participation|”decision making”|empowerment|”patient education”|”patient satisfaction”|”self efficacy”|”self management”

Years: 2015 to latest; Papers: 200

MEDLINE (EBSCOhost)

#	Query
S1	(MH "Perioperative Care") OR (MH "Perioperative Medicine") OR (MH "Perioperative Nursing") OR (MH "Perioperative Period") OR (MH "Postoperative Care") OR (MH "Postoperative Period") OR (MH "Preoperative Care") OR (MH "Preoperative Period")
S2	TI ((peri-operative OR perioperative OR post-operative OR postoperative OR post-surgery OR “post surgery” OR pre-operative OR preoperative OR pre-surgery OR surgery OR surgical) N3 (care OR medicine OR nurs* OR patient* OR period* OR procedure*)) OR AB ((peri-operative OR perioperative OR post-operative OR postoperative OR post-surgery OR “post surgery” OR pre-operative OR preoperative OR pre-surgery OR surgery OR surgical) N3 (care OR medicine OR nurs* OR patient* OR period* OR procedure*))
S3	S1 OR S2
S4	(MH "Telemedicine") OR (MH "Telenursing") OR (MH "Telerehabilitation") OR (MH "Mobile Applications") OR (MH "Cell Phone") OR (MH "Computers, Handheld") OR (MH "Smartphone") OR (MH "Electronic Mail") OR (MH "Text Messaging") OR (MH "Medical Informatics") OR (MH "Nursing Informatics")
S5	TI ((e-health OR ehealth OR m-health OR mhealth OR tele-health OR telehealth OR tele-medicine OR telemedicine OR tele-nursing OR telenursing OR tele-rehabilitation OR telerehabilitation) OR ((cell-phone OR cellphone* OR “cellular phone*” OR “electronic mail*” OR e-mail* OR email* OR “hand-held computer*” OR “handheld computer*” OR “hand-held device*” OR “handheld device*” OR iPad* OR iPhone* OR mobile* OR “palmtop computer*” OR “palmtop device*” OR PDA* OR “personal digital assistant*” OR smart-phone* OR smartphone* OR tablet* OR SMS OR MMS OR text-messag* OR “text messag*”)N3(app* OR application* OR program*)) OR ((health OR medical OR nursing) N3 informatics)) OR AB ((e-health OR ehealth OR m-health OR mhealth OR tele-health OR telehealth OR tele-medicine OR telemedicine OR tele-nursing OR telenursing OR tele-rehabilitation OR telerehabilitation) OR ((cell-phone OR cellphone* OR “cellular phone*” OR “electronic mail*” OR e-mail* OR email* OR “hand-held computer*” OR “handheld computer*” OR “hand-held device*” OR “handheld device*” OR iPad* OR iPhone* OR

	mobile* OR “palmtop computer*” OR “palmtop device*” OR PDA* OR “personal digital assistant*” OR smart-phone* OR smartphone* OR tablet* OR SMS OR MMS OR text-messag* OR “text messag*”)N3(app* OR application* OR program*)) OR ((health OR medical OR nursing) N3 informatics))
S6	S4 OR S5
S7	(MH "Self Care") OR (MH "Decision Making, Shared") OR (MH "Empowerment") OR (MH "Patient Education as Topic") OR (MH "Patient Participation") OR (MH "Patient Satisfaction") OR (MH "Self Efficacy") OR (MH "Self-Management")
S8	TI ((self-care OR self-efficacy OR self-manage* OR self-monitor*) OR (self N3 (care OR efficacy OR manage* OR monitor*)) OR (decision-making OR “decision making”) OR (decision* N3 (make OR making)) OR empower* OR ((patient* OR client* OR consumer*) N3 (education OR participation OR satisfaction)) OR (self-reported N3 (assessment* OR outcome*)) OR recovery) OR AB ((self-care OR self-efficacy OR self-manage* OR self-monitor*) OR (self N3 (care OR efficacy OR manage* OR monitor*)) OR (decision-making OR “decision making”) OR (decision* N3 (make OR making)) OR empower* OR ((patient* OR client* OR consumer*) N3 (education OR participation OR satisfaction)) OR (self-reported N3 (assessment* OR outcome*)) OR recovery)
S9	S7 OR S8
S10	S3 AND S6 AND S9

Limiters - Published Date: 20150101-20200631; Language: Danish, English, Norwegian, Swedish

PsycInfo (ProQuest)

(((MAINSUBJECT.EXACT("Surgery") OR MAINSUBJECT.EXACT("Surgical Patients")) OR (ti((peri-operative OR perioperative OR post-operative OR postoperative OR post-surgery OR "post surgery" OR pre-operative OR preoperative OR pre-surgery OR surgery OR surgical) NEAR/3 (care OR medicine OR nurs* OR patient* OR period* OR procedure*)) OR ab((peri-operative OR perioperative OR post-operative OR postoperative OR post-surgery OR "post surgery" OR pre-operative OR preoperative OR pre-surgery OR surgery OR surgical) NEAR/3 (care OR medicine OR nurs* OR patient* OR period* OR procedure*)))) AND ((MAINSUBJECT.EXACT("Mobile Health") OR MAINSUBJECT.EXACT("Telemedicine") OR MAINSUBJECT.EXACT("Telerehabilitation") OR MAINSUBJECT.EXACT("Mobile Applications") OR MAINSUBJECT.EXACT("Computer Mediated Communication") OR MAINSUBJECT.EXACT("Mobile Devices") OR MAINSUBJECT.EXACT("Mobile Phones") OR MAINSUBJECT.EXACT("Smartphones") OR MAINSUBJECT.EXACT("Text Messaging") OR MAINSUBJECT.EXACT("Health Information Technology")) OR (ti((e-health OR ehealth OR m-health OR mhealth OR tele-health OR telehealth OR tele-medicine OR telemedicine OR tele-nursing OR telenursing OR tele-rehabilitation OR telerehabilitation) OR ((cell-phone OR cellphone* OR "cellular phone*" OR "electronic mail*" OR e-mail* OR email* OR "hand-held computer*" OR "handheld computer*" OR "hand-held device*" OR "handheld device*" OR iPad* OR iPhone* OR mobile* OR "palmtop computer*" OR "palmtop device*" OR PDA* OR "personal digital assistant*" OR smart-phone* OR smartphone* OR tablet* OR SMS OR MMS OR text-messag* OR "text messag*") NEAR/3 (app* OR application* OR program*)) OR ((health OR medical OR nursing) NEAR/3 informatics)) OR ab((e-health OR ehealth OR m-health OR mhealth OR tele-health OR telehealth OR tele-medicine OR telemedicine OR tele-nursing OR telenursing OR tele-rehabilitation OR telerehabilitation) OR ((cell-phone OR cellphone* OR "cellular phone*" OR "electronic mail*" OR e-mail* OR email* OR "hand-held computer*" OR "handheld computer*" OR "hand-held device*" OR "handheld device*" OR iPad* OR iPhone* OR mobile* OR "palmtop computer*" OR "palmtop device*" OR PDA* OR "personal digital assistant*" OR smart-phone* OR smartphone* OR tablet* OR SMS OR MMS OR text-messag* OR "text messag*") NEAR/3 (app* OR application* OR program*)) OR ((health OR medical OR nursing) NEAR/3 informatics)))) AND ((MAINSUBJECT.EXACT("Client Education") OR MAINSUBJECT.EXACT("Client Participation") OR

MAINSUBJECT.EXACT("Client Satisfaction") OR MAINSUBJECT.EXACT("Decision Making") OR MAINSUBJECT.EXACT("Empowerment") OR MAINSUBJECT.EXACT("Self-Efficacy") OR MAINSUBJECT.EXACT("Self-Management")) OR (ti((self-care OR self-efficacy OR self-manage* OR self-monitor*) OR (self NEAR/3 (care OR efficacy OR manage* OR monitor*)) OR (decision-making OR "decision making") OR (decision* NEAR/3 (make OR making)) OR empower* OR ((patient* OR client* OR consumer*) NEAR/3 (education OR participation OR satisfaction)) OR (self-reported NEAR/3 (assessment* OR outcome*)) OR recovery) OR ab((self-care OR self-efficacy OR self-manage* OR self-monitor*) OR (self NEAR/3 (care OR efficacy OR manage* OR monitor*)) OR (decision-making OR "decision making") OR (decision* NEAR/3 (make OR making)) OR empower* OR ((patient* OR client* OR consumer*) NEAR/3 (education OR participation OR satisfaction)) OR (self-reported NEAR/3 (assessment* OR outcome*)) OR recovery)))) AND la.exact("Norwegian" OR "Swedish" OR "English" OR "Danish") AND PEER(yes) AND pd(20150101-20200602)

Web of Science Core Collection

Set	
# 4	(#1 AND #2 AND #3) *AND* LANGUAGE: (English OR Danish OR Norwegian OR Swedish)<br>*Indexes=SCI-EXPANDED, SSCI, A&HCI, CPCI-S, CPCI-SSH, ESCI Timespan=2015-2020*
# 3	TS = ((self-care OR self-efficacy OR self-manage* OR self-monitor*) OR (self NEAR/3 (care OR efficacy OR manage* OR monitor*)) OR (decision-making OR “decision making”) OR (decision* NEAR/3 (make OR making)) OR empower* OR ((patient* OR client* OR consumer*) NEAR/3 (education OR participation OR satisfaction)) OR (self-reported NEAR/3 (assessment* OR outcome*)) OR recovery)<br>*Indexes=SCI-EXPANDED, SSCI, A&HCI, CPCI-S, CPCI-SSH, ESCI Timespan=All years*
# 2	TS = ((e-health OR ehealth OR m-health OR mhealth OR tele-health OR telehealth OR tele-medicine OR telemedicine OR tele-nursing OR telenursing OR tele-rehabilitation OR telerehabilitation) OR ((cell-phone OR cellphone* OR “cellular phone*” OR “electronic mail*” OR e-mail* OR email* OR “hand-held computer*” OR “handheld computer*” OR “hand-held device*” OR “handheld device*” OR iPad* OR iPhone* OR mobile* OR “palmtop computer*” OR “palmtop device*” OR PDA* OR “personal digital assistant*” OR smart-phone* OR smartphone* OR tablet* OR SMS OR MMS OR text-messag* OR “text messag*”) NEAR/3(app* OR application* OR program*)) OR ((health OR medical OR nursing) NEAR/3 informatics))<br>*Indexes=SCI-EXPANDED, SSCI, A&HCI, CPCI-S, CPCI-SSH, ESCI Timespan=All years*
# 1	TS= ((peri-operative OR perioperative OR post-operative OR postoperative OR post-surgery OR “post surgery” OR pre-operative OR preoperative OR pre-surgery OR surgery OR surgical) NEAR/3 (care OR medicine OR nurs* OR patient* OR period* OR procedure*))<br>*Indexes=SCI-EXPANDED, SSCI, A&HCI, CPCI-S, CPCI-SSH, ESCI Timespan=All years*

Scopus (Elsevier)

(TITLE-ABS-KEY ((peri-operative OR perioperative OR post-operative OR postoperative OR post-surgery OR "post surgery" OR pre-operative OR preoperative OR pre-surgery OR surgery OR surgical) W/2 (care OR medicine OR nurs* OR patient* OR period* OR procedure*))) AND (TITLE-ABS-KEY ((e-health OR ehealth OR m-health OR mhealth OR tele-health OR telehealth OR tele-medicine OR telemedicine OR tele-nursing OR telenursing OR tele-rehabilitation OR telerehabilitation) OR ((cell-phone OR cellphone* OR "cellular phone*" OR "electronic mail*" OR e-mail* OR email* OR "hand-held computer*" OR "handheld computer*" OR "hand-held device*" OR "handheld device*" OR ipad* OR iphone* OR mobile* OR "palmtop computer*" OR "palmtop device*" OR pda* OR "personal digital assistant*" OR smart-

phone* OR smartphone* OR tablet* OR sms OR mms OR text-messag* OR "text messag*") W/2 (app* OR application* OR program*)) OR ((health OR medical OR nursing) W/2 informatics))) AND (TITLE-ABS-KEY ((self-care OR self-efficacy OR self-manage* OR self-monitor*) OR (self W/2 (care OR efficacy OR manage* OR monitor*)) OR (decision-making OR "decision making") OR (decision* W/2 (make OR making)) OR empower* OR ((patient* OR client* OR consumer*) W/2 (education OR participation OR satisfaction)) OR (self-reported W/2 (assessment* OR outcome*)) OR recovery)) AND PUBYEAR > 2014 AND NOT INDEX (medline) AND (LIMIT-TO (DOCTYPE , "ar"))
